# Lesion Location and Functional Connections Reveal Cognitive Impairment Networks in Multiple Sclerosis

**DOI:** 10.1002/acn3.70199

**Published:** 2025-10-05

**Authors:** Alessandro Franceschini, Paolo Preziosa, Paola Valsasina, Damiano Mistri, Monica Margoni, Federica Esposito, Massimo Filippi, Maria A. Rocca

**Affiliations:** ^1^ Neuroimaging Research Unit, Division of Neuroscience IRCCS San Raffaele Scientific Institute Milan Italy; ^2^ Neurology Unit IRCCS San Raffaele Scientific Institute Milan Italy; ^3^ Vita‐Salute San Raffaele University Milan Italy; ^4^ Neurorehabilitation Unit IRCCS San Raffaele Scientific Institute Milan Italy; ^5^ Neurophysiology Service IRCCS San Raffaele Scientific Institute Milan Italy

**Keywords:** cognitive impairment, fatigue, lesion network mapping, MRI, multiple sclerosis

## Abstract

**Objective:**

Cognitive impairment, fatigue, and depression are common in multiple sclerosis (MS), potentially due to disruption of regional functional connectivity caused by white matter (WM) lesions. We explored whether WM lesions functionally connected to specific brain regions contribute to these MS‐related manifestations.

**Methods:**

A total of 596 MS patients underwent 3T brain MRI acquisition, neurologic assessment, and neuropsychological evaluation (Brief Repeatable Battery, Modified Fatigue Impact Scale [MFIS], and Montgomery‐Åsberg Depression Rating Scale [MADRS]). Voxel‐wise lesion probability maps were compared between subgroups based on cognition, fatigue, or depression. Lesion distributions were linked to a brain functional connectivity atlas to map lesion network associations. Lesion network maps (LNMs) were then compared among subgroups (*p* < 0.05, FWE‐corrected).

**Results:**

One hundred twenty‐six (27.2%) MS patients were cognitively impaired and showed significantly more widespread WM lesions, more strongly functionally connected to bilateral hippocampi, thalami, cerebellum, and occipital cortices (corrected‐*p* < 0.05) than cognitively preserved patients. Lesion networks were similar for impaired processing speed/attention. Verbal memory deficits were associated with WM lesions connected to parahippocampi, temporal pole, and cerebellum (corrected‐*p* ≤ 0.05), while verbal fluency deficits involved connections to thalami, putamen, caudate nuclei, anterior cingulate cortex, and cerebellum (corrected‐*p* ≤ 0.05). No significant lesion distribution or network connectivity differences were found in patients with visual memory deficits, fatigue (MFIS ≥ 38, 184/493 [37.3%]) or depression (MADRS > 9, 192/495 [38.8%]).

**Interpretation:**

Regional WM lesions disrupting connections to the hippocampus, thalamus, cerebellum, and temporo‐occipital cortices contribute to cognitive impairment, but not fatigue or depression. LNM may clarify mechanisms underlying cognitive deficits in MS.

## 1 Introduction

Multiple sclerosis (MS) is a chronic immune‐mediated disease of the central nervous system (CNS) characterized by inflammation, demyelination, and neurodegeneration. Beyond locomotor symptoms, MS often presents with cognitive impairment, fatigue, and depression, which substantially impact patients' quality of life [1, 2].

Cognitive impairment affects up to 70% of MS patients [2] and may result from complex mechanisms [1, 2], including microstructural abnormalities in normal‐appearing white matter (NAWM), gray matter (GM) atrophy, and progressive exhaustion of adaptive brain functional network processes [2, 3]. White matter (WM) disconnection is also crucial, as it disrupts integration among brain regions relevant to cognition [1, 2]. Brain T2‐hyperintense WM lesions, particularly within cognitively relevant WM tracts, have been associated with cognitive dysfunction, though findings have been inconsistent [2, 3, 4]. Fatigue, affecting up to 81% of MS patients, especially in progressive forms of the disease [5], has been associated with abnormal functional connectivity in fronto‐parietal cortices and basal ganglia [5, 6, 7, 8]. Multiparametric MRI studies also support the role of structural damage to anterior cortico‐subcortical networks in the pathogenesis of fatigue, although with conflicting findings [5, 6, 7, 8]. Depression, reported in up to 50% of MS patients [1], has been associated with structural and functional abnormalities involving the medial temporal lobe (i.e., hippocampus and amygdala), medial prefrontal cortex, and parietal cortices [9, 10, 11, 12].

However, similar clinical manifestations may arise from T2‐hyperintense WM lesions in anatomically distinct regions, likely due to remote effects on interconnected areas through disconnection or diaschisis, complicating symptom localization. By mapping intrinsic functional brain connections disrupted by T2‐hyperintense WM lesions using a large, normative human brain connectome [13], lesion network mapping (LNM) has been proposed as a method to explore how lesions with heterogeneous sizes and topographies may lead to similar clinical symptoms through shared connectivity patterns with specific brain regions [14, 15]. LNM has identified disease‐specific networks in stroke, small vessel disease, and psychiatric disorders [16, 17, 18], and, recently, in MS [19, 20], where memory‐related lesions mapped to a hippocampus‐centered circuit [19], overlapping with a stroke‐derived memory circuit [21]. However, that study lacked a comprehensive assessment of cognitive functions. A recent study associated the resting‐state (RS) functional connectivity (FC) of T2‐hyperintense WM lesions with the ventral midbrain to depression severity in MS patients, based on an a priori depression circuit derived from stroke and neuromodulation studies [20]. Yet, the analysis relied on a predefined template and was restricted to depression. To date, LNM has not been applied to explore lesion‐fatigue associations in MS.

In this study, we comprehensively investigated the associations between T2‐hyperintense WM lesions and their RS FC profiles with cognitive impairment, fatigue, and depression in a large cohort of MS patients. Specifically, we explored whether specific locations of T2‐hyperintense WM lesions and the FC of those lesions to strategic brain regions may contribute to these MS‐related manifestations.

## 2 Methods

### 2.1 Standard Protocol Approvals, Registrations, and Patient Consents

The institutional ethical standards committee on human experimentation at IRCCS Ospedale San Raffaele approved experiments involving human subjects (Protocol N° 2009–74). Before participating in the study, all subjects provided written informed consent in accordance with the Declaration of Helsinki.

### 2.2 Study Population

We retrospectively identified 596 MS patients from the Neuroimaging Research Unit database at IRCCS Ospedale San Raffaele, Milan, who underwent clinical, neuropsychological, and MRI evaluations between November 2009 and January 2024. Inclusion criteria were: age ≥ 18 years, a diagnosis of MS according to the 2017 revision of the McDonald criteria, native Italian language, no relapses or steroid use within 3 months prior to MRI, absence of significant neurologic (other than MS) or psychiatric comorbidities affecting cognition, absence of secondary fatigue based on clinical medical history, and stable disease‐modifying treatment for at least 6 months.

### 2.3 Neurologic and Neuropsychological Assessment

Experienced neurologists, blinded to MRI results, performed a neurologic examination, rated the Expanded Disability Status Scale (EDSS) score, and defined the MS clinical phenotype (relapsing–remitting or progressive) within 3 days from MRI acquisition. The Brief Repeatable Battery of Neuropsychological Tests (BRB‐N) [22] was administered to all MS patients on the day of MRI acquisition to assess cognitive function across four domains: verbal memory (Selective Reminding Test‐ [SRT] long‐term storage, SRT‐consistent long‐term retrieval, SRT‐delayed recall [22]), visual memory (10/36 Spatial Recall Test [SPART] and SPART delayed recall [SPART‐D] [22]), information processing speed/attention (symbol digit modalities test [SDMT] [22], paced auditory serial addition test [PASAT] with a rate of number presentation of 3 [PASAT‐3] and 2 s [PASAT‐2] [22]), and verbal fluency with the Word List Generation (WLG) [22]. Z‐scores for all BRB‐N tests were computed using regression models adjusted for sex, age, and education, based on a recent updated Italian normative dataset [23]. Domain‐specific z‐scores were then calculated by averaging z‐scores of tests within those cognitive domains, as previously described [24]. Finally, a global z‐score for cognitive functions was obtained by averaging z‐scores of cognitive domains. Test failure was defined as a performance at least 1.5 standard deviations below normative values [25]. Impairment in a single domain was defined as failure in at least one test assessing that domain, whereas patients with at least one abnormal neuropsychological test in two or more cognitive domains were classified as cognitively impaired [25].

Fatigue was assessed using the Modified form of the Fatigue Impact Scale (MFIS), a validated comprehensive self‐report tool designed to evaluate the impact of fatigue on physical, cognitive, and psychosocial functioning [26]. Patients with an MFIS score ≥ 38 were classified as fatigued [27].

Depressive symptoms were assessed using the Montgomery‐Åsberg Depression Rating Scale (MADRS) [28]. Patients with a MADRS score > 9 were classified as depressed [29].

Accordingly, patients were grouped for analysis based on cognitive impairment as well as the presence of fatigue or depressive symptoms. A single patient could be included in more than one analysis group if they met the corresponding criteria.

### 2.4 MRI Acquisition

For MRI acquisition, we used two 3.0T scanners (Scanner 1: Achieva, Philips Medical Systems, Eindhoven, The Netherlands: 305 MS patients; Scanner 2: Ingenia, Philips Medical systems, Eindhoven, The Netherlands: 291 MS patients). On Scanner 1, we acquired: (1) dual‐echo (DE) turbo spin echo sequence (repetition time [TR] = 2599 ms; echo time [TE] = 16–80 ms; flip angle = 90°; matrix = 256 × 256; field of view [FOV] = 240 × 240 mm^2^; echo train length [ETL] = 6; 44 contiguous axial slices, 3‐mm thick) and (2) three‐dimensional (3D) T1‐weighted fast field echo sequence (TR = 25; TE = 4.6 ms; flip angle = 30°; matrix = 256 × 256; FOV = 230 mm^2^; 220 contiguous axial slices, 0.8 mm thick). Images acquired using Scanner 2 included: (1) a 3D fluid attenuated inversion recovery (FLAIR) (TR = 4800 ms; TE = 270 ms; TI = 1650 ms; matrix size = 256 × 256; FOV = 256 × 256 mm^2^; ETL = 167; 192 contiguous sagittal slices, 1‐mm thick) and (2) a 3D T1‐weighted turbo field echo sequence (TR = 7 ms; TE = 3.2 ms; TI = 1000 ms; flip angle = 8°; matrix size = 256 × 256; FOV = 256 × 256 mm^2^; 204 contiguous sagittal slices, 1‐mm thick).

### 2.5 Conventional MRI Analysis

For MS patients acquired on Scanner 1, T2‐hyperintense WM lesion volume (LV) was quantified on DE images using a semi‐automatic local thresholding segmentation technique (Jim 7.0, Xinapse Systems Ltd., Colchester, UK). For patients acquired on Scanner 2, T2‐hyperintense WM LV was quantified by using a fully automated pipeline based on a cascade of two 3D patch‐wise convolutional neural networks. This approach utilized 3D FLAIR and 3D T1‐weighted MRI sequences as input images [30], followed by visual quality control and manual editing, if necessary. For patients, regardless of scanner, normalized brain volume was calculated using FSL SIENAx software on lesion‐filled 3D T1‐weighted images.

### 2.6 T2‐Hyperintense Lesion Probability Mapping (LPM)

**FIGURE 1:**
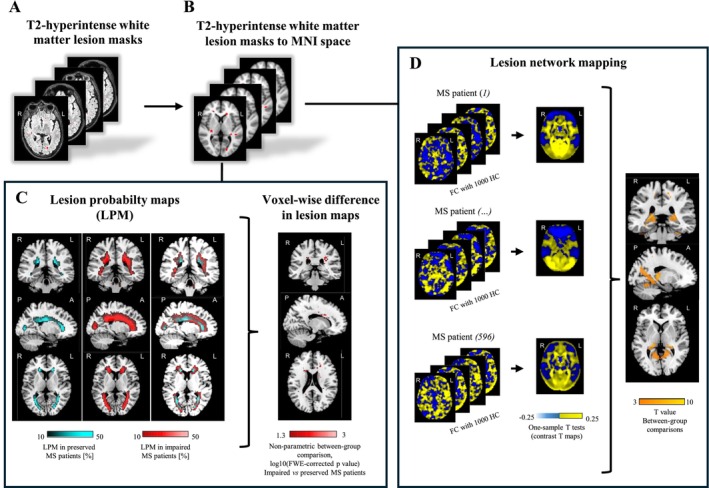
Schematic representation of study methodology to explore between‐group differences in T2‐hyperintense white matter lesion probability maps (LPMs) and lesion network mapping (LNM) according to cognitive performance, as well as fatigue and depression status. (A) T2‐hyperintense white matter lesion masks of multiple sclerosis (MS) patients constitute the starting point for MRI analysis; (B) Binary T2‐hyperintense white matter lesion masks are co‐registered to standard Montral Neurological Institute (MNI) space; (C) lesion masks of cognitively preserved and impaired MS patients are averaged to obtain T2‐hyperintense white matter LPMs, colored in green for preserved and in red for impaired MS patients, and are compared in a voxel‐wise manner between impaired and preserved MS patients (Statistical non‐Parametric Mapping toolbox within SPM12 software, non‐parametric comparison between two groups adjusted for scanner, age, sex and EDSS score, 5000 permutations, *p* < 0.05 family‐wise error corrected for multiple comparisons); (D) LNM procedure, which utilizes lesion masks of MS patients as seeds for resting state functional connectivity (RS FC) analysis, computed on pre‐processed RS functional MRI data from a publicly available large cohort of 1000 healthy controls (HC) acquired for the brain GSP [13]. For each subject included in the GSP database, the mean RS functional MRI time course is extracted from lesion masks, correlated with the time series of any other brain voxel (positive correlations in yellow and negative in blue), and Fisher's z transformed to improve Gaussianity. RS FC maps obtained for each lesion mask from the 1000 HC are then averaged (by means of SPM12 one‐sample *t*‐test). Corresponding T maps are LNMs, which undergo between‐group comparison in a voxel‐wise manner (e.g., impaired vs. preserved MS patients) using SPM12 (full factorial model adjusted for scanner, age, sex and EDSS score, *p* < 0.05 cluster‐wise family‐wise error corrected for multiple comparisons). A, anterior; EDSS, Expanded Disability Status Scale; FC, functional connectivity; GSP, Genomic Superstruct Project; HC, healthy controls; L, left; LNM, lesion network mapping; LPM, lesion probability map; MNI, Montreal Neurological Institute; MS, multiple sclerosis; P, posterior; R, right; RS, resting state; SnPM, statistical non‐parametric mapping; SPM, statistical parametric mapping.

DE/FLAIR images were first rigidly co‐registered to 3D T1‐weighted scans of the same subject using FSL FLIRT. The resulting transformation was then applied to binarized T2‐hyperintense lesion masks. Then, using a combination of FSL FLIRT and FNIRT, 3D T1‐weighted scans of each subject were co‐registered to the Montreal Neurological Institute (MNI) space. The same transformation was applied to binarized T2‐hyperintense lesion masks, allowing them to be transformed to the standard space. Normalized masks were then averaged across different groups of patients stratified by cognitive performance, as well as fatigue and depression status (see statistical analysis for a detailed list of between‐group comparisons performed in the study) to obtain mean lesion probability maps (Figure 1).

### 2.7 T2‐Hyperintense Lesion Network Mapping (LNM)

LNM was implemented using a method described in previous publications [14, 15, 16, 20, 31]. Briefly, normalized T2‐hyperintense WM lesion masks were used as seeds for RS FC analysis, which was computed on pre‐processed RS functional MRI data from a publicly available large cohort of 1000 healthy subjects acquired for the brain Genomic Superstruct Project (GSP) [13]. For each subject included in the GSP database, the mean RS functional MRI time course was extracted from lesion masks, correlated with the time series of any other brain voxel, and Fisher's r‐to‐z transformed to improve Gaussianity. RS FC maps obtained for each lesion mask from the 1000 healthy subjects were then combined (by means of SPM12 one‐sample *t*‐test) to obtain the average RS FC profile of each lesion mask. The corresponding contrast T maps constitute lesion network maps and represent maps of regions functionally connected to each patient's lesion location. Such maps were used as inputs for subsequent between‐group comparisons (Figure 1).

### 2.8 Statistical Analysis

Differences in demographic and clinical variables between MS patients according to the presence of cognitive impairment, fatigue, and depression were compared using Chi‐square, two‐sample *t*, and Mann–Whitney *U* tests, as appropriate. Between‐group comparisons of T2‐hyperintense LV and normalized brain volume across the same groups were assessed using age‐, sex‐, and scanner‐adjusted ANOVA models. T2‐hyperintense WM LVs were log‐transformed. Moreover, we used the Spearman's rank correlation coefficient to assess possible associations among clinical, cognitive, depression, and fatigue scores. A *p*‐value < 0.05 was considered statistically significant. SPSS (IBM SPSS Statistics, version 26.0) was used for such analyses.

Lesion masks were compared between different groups using the Statistical non‐Parametric Mapping (SnPM) toolbox included in SPM12, using age‐, sex‐, EDSS score‐, and scanner‐adjusted nonparametric models with 5000 permutations, while LNMs were compared between different groups using SPM12 and two‐sample *t*‐tests including as confounding covariates age, sex, EDSS score, T2 LV, and acquisition scanner (Figure 1). We chose to correct all statistical models for EDSS score because of its possible confounding effect on cognition, depression, and fatigue (in other words, because cognitively impaired/fatigued/depressed patients tended to have greater disability than cognitively preserved/non‐fatigued/non‐depressed patients). The following comparisons between MS patients' groups were performed: (1) cognitively preserved versus cognitively impaired; (2) information processing speed/attention preserved versus impaired; (3) verbal memory preserved versus impaired; (4) verbal fluency preserved versus impaired; (5) visual memory impaired versus preserved; (6) fatigued versus non‐fatigued; and (7) depressed versus non‐depressed. Results were considered statistically significant if surviving at *p* < 0.05, cluster‐wise family‐wise error (FWE) corrected for multiple comparisons (cluster‐forming threshold: *p* < 0.001, cluster extent k_E_ = 50 voxels). To limit consequences of partial volume effects, all results derived from lesion‐mask comparisons were explicitly masked with a white matter mask, obtained by thresholding a white matter probability atlas in the standard space (available in SPM12 software) at 0.25. Conversely, all results derived from LNM comparisons were explicitly masked with a gray matter mask, obtained by thresholding a gray matter probability atlas in the standard space (available in SPM12 software) at 0.25. The location of clusters deriving from SPM analyses was determined by using the JHU_WM tractography atlas for lesion masks and the Automatic Anatomical Labelling (AAL) atlas for LNMs.

### 2.9 Validation Analysis

The use of two different imaging sequences and segmentation techniques on Scanner 1 and Scanner 2 for lesion assessment may risk introducing heterogeneity in the analysis and may potentially bias the results. To test whether such a confounding effect was present, we produced mean LPM for Scanner 1 and Scanner 2, separately, in cognitively preserved, cognitively impaired, non‐fatigued, fatigued, non‐depressed, and depressed MS patients. Then, to quantify lesion probability map similarity, we calculated the Dice coefficient and the mean square error (MSE) as metrics of similarity between Scanner 1 and Scanner 2 LPMs in the above‐defined groups.

## 3 Results

### 3.1 Demographic, Clinical and Conventional MRI Features of MS Patients

The study population included 596 MS patients (mean age = 43.7 years, standard deviation [SD] = 11.5; 350 women), with a mean disease duration of 13.1 years (SD = 9.8) and a median EDSS score of 2.5 (interquartile range [IQR = 1.5;5.5]). Three hundred eighty‐three (64%) MS patients had a relapsing–remitting course, whereas 213 (36%) had a progressive phenotype. See Table S1 for additional details.

In our study population, 162 out of 596 (27.2%) patients were classified as cognitively impaired (Table 1). The most frequently affected cognitive domains were information processing speed/attention (81% of cognitively impaired patients), verbal memory (78% of cognitively impaired), visual memory (54% of cognitively impaired patients), and verbal fluency (48% of cognitively impaired patients) (Table 2).

**TABLE 1:** Demographic, clinical, and MRI features of cognitively impaired vs. preserved multiple sclerosis (MS) patients.

Variables	Cognitively preserved MS patients (*n* = 434)	Cognitively impaired MS patients (*n* = 162)	*p*
Sex (%): women/men	262 (60)/172 (40)	88 (54)/74 (46)	0.182^a^
Mean age (SD) [years]	42.8 (11.3)	46.2 (11.5)	**0.001** ^b^
Scanner 1/2	237/197	68/94	**0.004** ^a^
Mean disease duration (SD) [years]	12.3 (9.6)	15.5 (9.9)	**< 0.001** ^b^
Median EDSS score (IQR)	2.0 (1.5;5.0)	4.5 (2.0;6.0)	**< 0.001** ^c^
Clinical phenotype (%): RRMS/PMS	305 (70)/129 (30)	78 (48)/84 (52)	**< 0.001** ^a^
Ongoing DMT (%): none/first line/second line^d^	85 (20)/184 (42)/165 (38)	49 (30)/58 (36)/55 (34)	**0.021** ^a^
Median education (IQR) [years]	13.0 (9.3;17.8)	13.0 (8.1;17.2)	0.631^c^
Mean MFIS (SD)^e^	29.7 (16.5)	39.8 (19.1)	**< 0.001** ^b^
Mean MADRS (SD)^f^	9.0 (7.7)	9.9 (7.2)	0.296^b^
Median T2‐ hyperintense LV (IQR) [mL]^g^	3.3 (1.2;7.2)	10.0 (3.6;19.6)	**< 0.001** ^h^
Estimated mean NBV (SE) [mL]	1538 (3)	1492 (5)	**< 0.001** ^h^

*Note:* Bold text indicates a statistically significant result.

Abbreviations: DMT, disease‐modifying therapy; EDSS, Expanded Disability Status Scale; IQR, interquartile range; LV, lesion volume; MADRS, Montgomery‐Åsberg Depression Rating Scale; MFIS, Modified form of the Fatigue Impact Scale; ml, milliliters; MS, multiple sclerosis; NBV, normal brain volume; PMS, progressive multiple sclerosis; RRMS, relapsing–remitting multiple; SD, standard deviation; SE, standard error.

^a^ Chi‐square test.

^b^ Two sample *t*‐test.

^c^ Mann‐Whitney *U* test.

^d^ First line = glatiramer acetate, interferon beta 1a, teriflunomide, or dimethyl fumarate; Second line = fingolimod, siponimod, ozanimod, natalizumab, cladribine, anti‐CD20 (ocrelizumab, rituximab, ofatumumab) and other immunosuppressants.

^e^ Available for 493 out of 596 MS patients.

^f^ Available for 495 out of 596 MS patients.

^g^ Comparison performed on log‐scale.

^h^ Age‐adjusted, scanner‐adjusted, and sex‐adjusted linear regression model.

**TABLE 2:** Mean z‐scores and prevalence of impairment at neuropsychological tests and at each cognitive domain explored by the Brief Repeatable Battery (BRB‐N) in multiple sclerosis (MS) patients according to cognitive status.

BRB‐N test	Cognitively preserved MS patients (*n* = 434)		Cognitively impaired MS patients (*n* = 162)		Cognitive domains	Cognitively preserved MS patients (*n* = 434)		Cognitively impaired MS patients (*n* = 162)	
	Z‐score^a^	*N* of impaired^b^ (%)	Z‐scoreª	*N* of impaired^b^ (%)		Z‐score^a^	*N* of impaired^c^ (%)	Z‐score^a^	*N* of impaired^c^ (%)
SRT lts	−0.04 (0.96)	26 (6)	−1.59 (1.00)	92 (57)	Verbal memory	−0.06 (0.96)	54 (12)	−1.60 (0.90)	126 (78)
SRT cltr	−0.12 (1.08)	41 (10)	−1.83 (1.04)	110 (68)					
SRT recall	0.06 (1.09)	20 (5)	−1.36 (1.00)	69 (43)					
SPART	0.22 (1.00)	8 (2)	−1.04 (0.95)	51 (44)	Visual memory	0.24 (0.94)	14 (3)	−1.08 (0.94)	88 (54)
SPART recall	0.26 (1.05)	10 (2)	−1.12 (1.21)	62 (40)					
SDMT	−0.09 (0.99)	28 (7)	−1.53 (1.00)	83 (52)	Information processing speed/attention	−0.14 (0.90)	81 (19)	−1.61 (0.91)	131 (81)
PASAT 3″	−0.04 (1.09)	39 (10)	−1.53 (1.07)	83 (58)					
PASAT 2″	−0.17 (1.13)	46 (12)	−1.61 (1.10)	68 (58)					
WLG	−0.03 (0.97)	23 (6)	−1.24 (1.04)	74 (48)	Verbal fluency	−0.03 (0.97)	23 (6)	−1.24 (1.04)	74 (48)
					Global cognition	−0.01 (0.61)		−1.40 (0.54)	

Abbreviations: BRB‐N = Brief Repeatable Battery of Neuropsychological Tests; cltr = consistent longterm retrieval; lts = long‐term storage; PASAT = paced auditory serial attention test; SDMT = symbol digit modalities test; SPART = spatial recall test; SRT = selective reminding test; WLG = word list generation.

^a^ Mean (SD) of z‐scores according to an update of Italian normative data from the Italian Neuroimaging Network Initiative (INNI) [23].

^b^ Number of patients (frequency) with an abnormal performance, defined as a score 1.5 SD below normative values according to the normative data of an Italian representative sample [25].

^c^ Number of patients (frequency) with ≥ 1 abnormal neuropsychological tests of BRB‐N for each cognitive domain.

Compared to cognitively preserved, cognitively impaired patients were significantly older (*p* = 0.001), had longer disease duration (*p* < 0.001), higher EDSS score (*p* < 0.001), higher MFIS score (*p* < 0.001), were more frequently progressive MS (*p* < 0.001), and were more frequently untreated (*p* = 0.021) (Table 1). Cognitively impaired patients also had higher T2‐hyperintense WM LV (*p* < 0.001) and lower normalized brain volume (*p* < 0.001) (Table 1).

MFIS score was available for 493 out of 596 (82.7%) patients. Among them, 184 out of 493 (37.3%) patients were classified as fatigued (Table S2). Compared to non‐fatigued patients, those with fatigue were significantly older (*p* < 0.001), had longer disease duration (*p* = 0.005), higher EDSS score (*p* < 0.001), higher MADRS score (*p* < 0.001), and were more frequently progressive MS (*p* < 0.001) (Table S2). Fatigued patients also had higher T2‐hyperintense WM LV (*p* < 0.001) and lower normalized brain volume (*p* = 0.005) (Table S2).

MADRS score was available for 495 out of 596 (83.1%) patients. Among them, 192 out of 495 (38.8%) patients were classified as depressed (Table S2). Compared to patients without depression, those with depression were more frequently women (*p* = 0.023), had significantly higher EDSS score (*p* < 0.001), higher MFIS score (*p* < 0.001), and were more frequently progressive MS (*p* = 0.032) (Table S2). T2‐hyperintense WM LV and normalized brain volume were not significantly different between patients with and without depression (*p* ≥ 0.076) (Table S2).

The EDSS score was moderately correlated with all cognitive scores (*r* ranging from −0.20 to −0.25, all *p* < 0.001), as well as with MFIS (*r* = 0.47, *p* < 0.001) and MADRS (*r* = 0.25, *p* < 0.001) scores. Disease duration was weakly associated with all cognitive scores (*r* ranging from −0.11 to −0.23, *p* ranging from 0.006 to < 0.001) and with MFIS (*r* = 0.17, *p* < 0.001), but not with MADRS (*r* = 0.08, *p* = 0.06). Finally, weak‐to‐moderate correlations were found between MFIS and cognitive scores (*r* ranging from −0.19 to −0.32, all *p* < 0.001), as well as between MADRS and cognitive scores (*r* ranging from −0.10 to −0.20, *p* ranging from 0.02 to < 0.001).

### 3.2 T2‐Hyperintense WM LPM and LNM According to Cognitive Performance

#### 3.2.1 Global Cognitive Function

**FIGURE 2:**
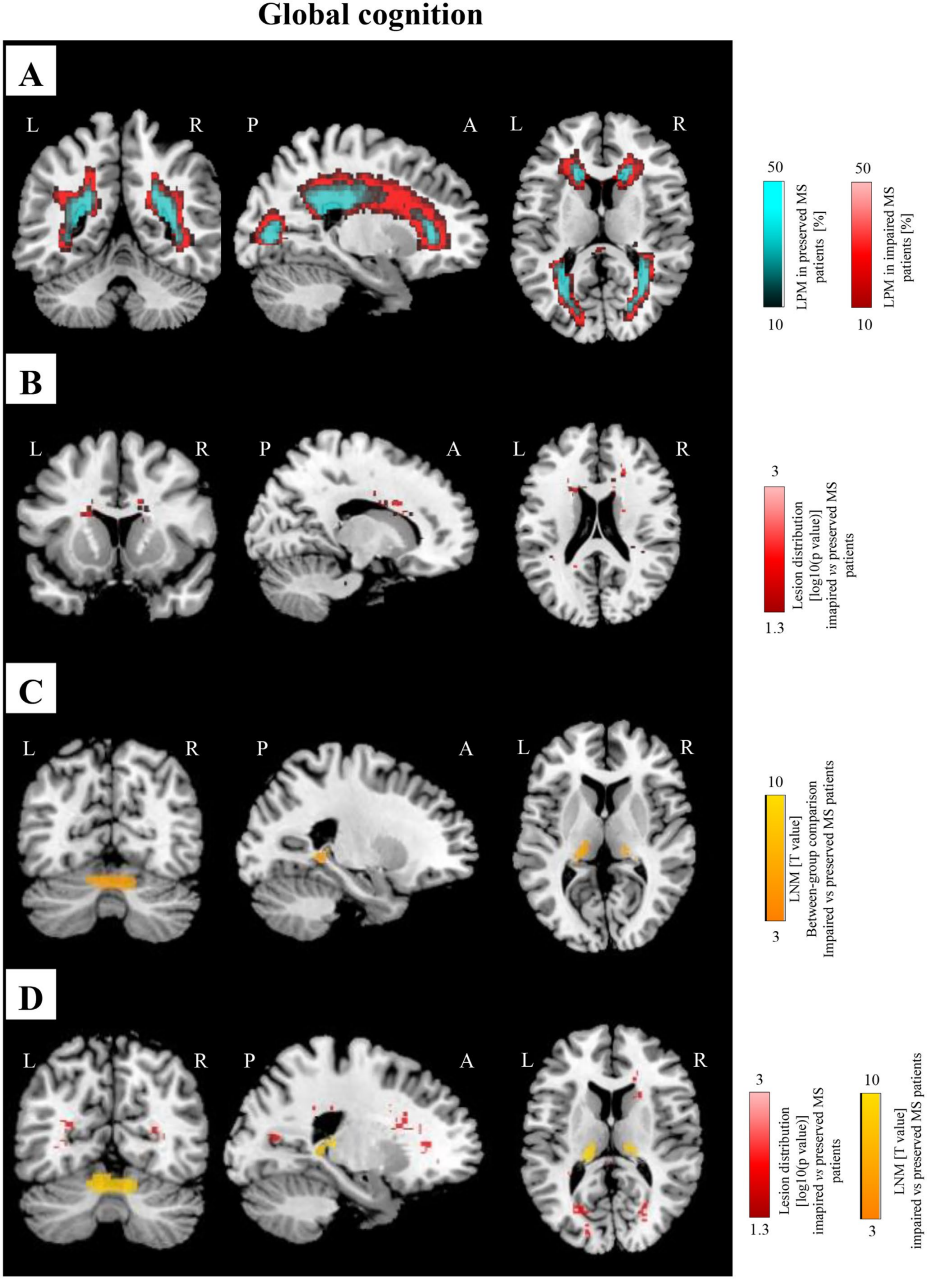
LPM and LNM findings according to performance in global cognitive function. (A) LPMs of cognitively impaired (in red, *N* = 162) and preserved (in cyan, *N* = 434) MS patients. (B) Difference of lesion distribution between cognitively impaired and preserved MS patients (in red, SnPM non parametric between‐group comparison). (C) Difference of LNMs between cognitively impaired and preserved MS patients (in orange, SPM12 two‐sample *t*‐test). (D) Overlay of the differences in LNM (in orange) and LPM (in red) between cognitively impaired and preserved MS patients, for a better highlight of the results provided by both methods. All analyses report results surviving at *p* < 0.05, FWE corrected for multiple comparisons (cluster‐forming threshold: *p* < 0.001, cluster extent *k*_E_ = 50). A, anterior; FWE, family‐wise error; L, left; LNM, lesion network map; LPM, lesion probability map; MS, multiple sclerosis; P, posterior; R, right; SnPM, statistical non‐parametric mapping; SPM, statistical parametric mapping.

Compared to cognitively‐preserved patients, those classified as cognitively impaired had a significantly higher prevalence of T2‐hyperintense WM lesions in the body of the corpus callosum, the superior and inferior longitudinal fasciculus, the anterior and posterior corona radiata, as well as the posterior thalamic radiation (*p* < 0.001, FWE corrected) (Figure 2; Table S3).

Compared to T2‐hyperintense WM lesions of cognitively preserved patients, those of cognitively impaired individuals were more functionally connected to bilateral hippocampi, thalami, and cerebellum (right and left lobule VI), as well as to the left lingual gyrus (*p* = 0.05, cluster‐wise FWE corrected) (Figure 2; Table S4). No significant results were detected for the opposite comparison, neither for T2‐hyperintense WM LPM nor for LNM.

#### 3.2.2 Information Processing Speed/Attention

**FIGURE 3:**
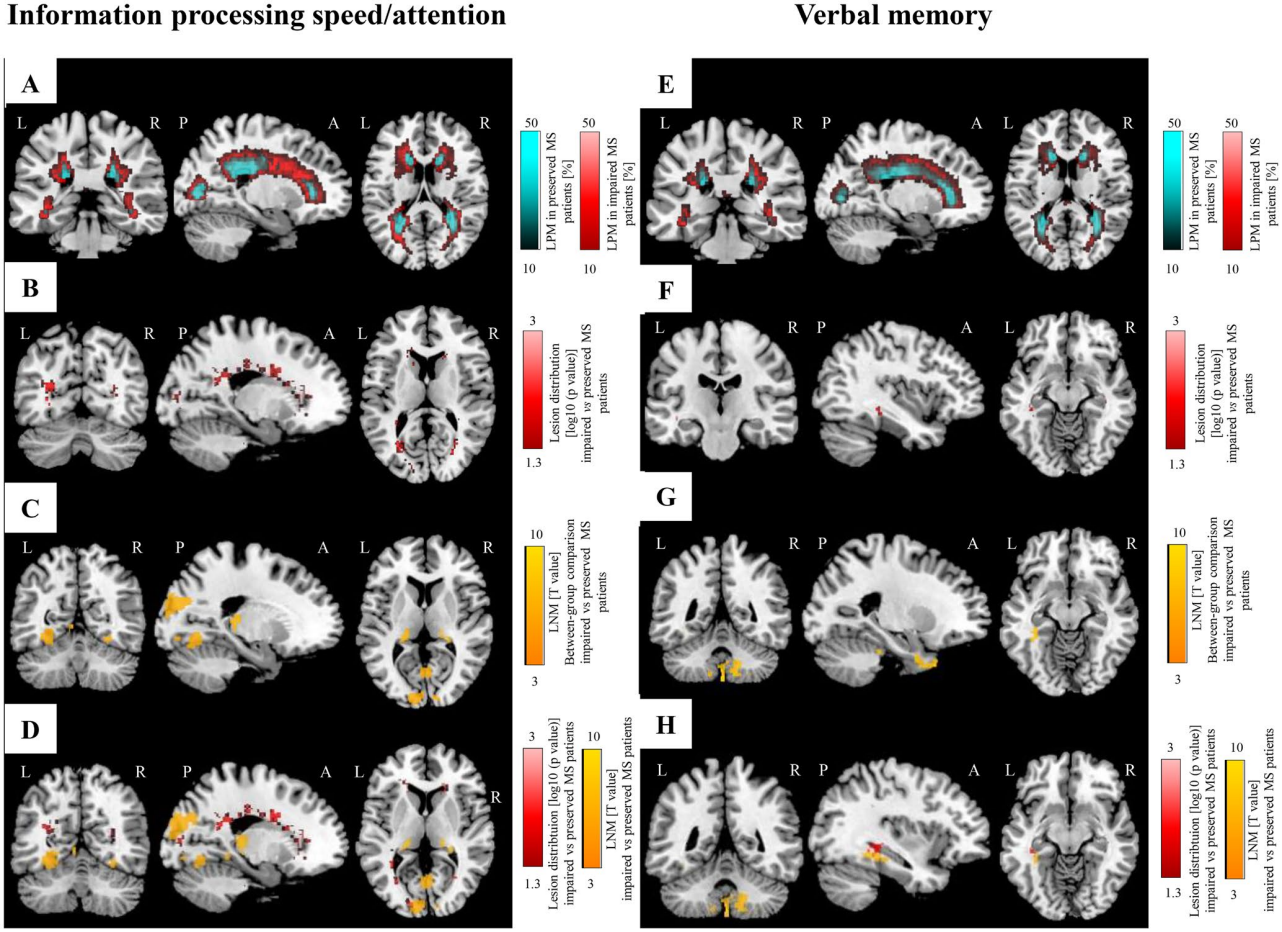
LPM and LNM findings according to performance in information processing speed/attention and verbal memory. (A) LPMs of MS patients impaired (in red, *N* = 212) and preserved (in cyan, *n* = 384) at information processing speed/attention. (B) Difference of lesion distribution between MS patients impaired and preserved at information processing/attention (in red, SnPM non parametric between‐group comparison). (C) Difference of LNMs between MS patients impaired and preserved at information processing speed/attention (in orange, SPM12 two‐sample *t*‐test). (D) Overlay of the differences in LNM (in orange) and LPM (in red) between MS patients impaired and preserved at information processing speed/attention, for a better highlight of the results provided by both methods. (E) LPMs of MS patients impaired (in red, *N* = 180) and preserved (in cyan, *N* = 416) at verbal memory. (F) Difference of lesion distribution between MS patients impaired and preserved at verbal memory (in red, SPM12 two‐sample *t*‐test). (G) Difference of LNMs between MS patients impaired and preserved at verbal memory (in orange, SPM12 two‐sample *t*‐test). (H) Overlay of the differences in LNM (in orange) and LPM (in red) between MS patients impaired and preserved at verbal memory, for a better highlight of the results provided by both methods. All analyses report results surviving at *p* < 0.05, FWE corrected for multiple comparisons (cluster‐forming threshold: *p* < 0.001, cluster extent *k*_E_ = 50). A, anterior; FWE, family‐wise error; L, left; LNM, lesion network map; LPM, lesion probability map; MS, multiple sclerosis; P, posterior; R, right; SnPM, statistical non‐parametric mapping; SPM, statistical parametric mapping.

Compared to preserved patients, those with impaired information processing/attention speed showed a higher prevalence of T2‐hyperintense WM lesions in the body, genu, and splenium of the corpus callosum, in the corona radiata, superior fronto‐occipital fasciculus, inferior longitudinal fasciculus, and posterior thalamic radiation (*p* < 0.001, FWE corrected) (Figure 3; Table S5).

Compared to T2‐hyperintense WM lesions of preserved patients, those of MS patients showing impaired information processing speed/attention were more functionally connected to several GM areas mainly located in posterior brain regions, including bilateral thalami, hippocampi, fusiform gyri, and lingual gyri, as well as to the right parahippocampal gyrus (*p* ≤ 0.05, cluster‐wise FWE corrected) (Figure 3; Table S6). No significant results were detected for the opposite comparison, neither for T2‐hyperintense WM LPM nor for LNM.

#### 3.2.3 Verbal Memory

Compared to preserved patients, those with impaired verbal memory had a higher prevalence of T2‐hyperintense WM lesions in a cluster localized in the left cingulum (*p* = 0.0008, FWE corrected) (Figure 3; Table S7).

Compared to T2‐hyperintense WM lesions of preserved patients, those showing impaired verbal memory were more functionally connected to several bilateral cerebellar regions (including lobules I‐IV, V and VI), bilateral temporal pole, and left parahippocampal gyrus (*p* = 0.05, cluster‐wise FWE corrected) (Figure 3; Table S8). No significant results were detected for the opposite comparison, neither for T2‐hyperintense WM LPM nor for LNM.

#### 3.2.4 Verbal Fluency

**FIGURE 4:**
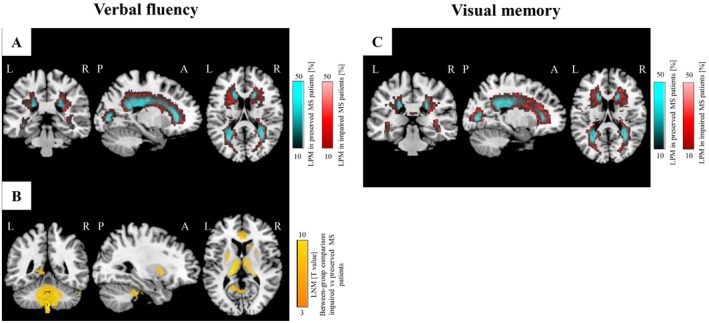
LPM and LNM findings according to performance in verbal fluency and visual memory. (A) LPMs of MS patients impaired (in red, *N* = 97) and preserved (in cyan, *N* = 471) at verbal fluency. (B) Differences of LNMs between MS patients impaired and preserved at verbal fluency (in orange, SPM12 two‐sample *t*‐test). No differences of lesion distribution between the same patients' group were detected. (C) LPMs of MS patients impaired (in red, *N* = 102) and preserved (in cyan, *N* = 494) at visual memory. No differences of lesion distribution and LNMs between MS patients impaired and preserved at visual memory. All analyses report results surviving at *p* < 0.05, FWE corrected for multiple comparisons (cluster‐forming threshold: *p* < 0.001, cluster extent *k*_E_ = 50). A, anterior; FWE, family‐wise error; L, left; LNM, lesion network map; LPM, lesion probability map; MS, multiple sclerosis; P, posterior; R, right; SPM, statistical parametric mapping.

No significant differences were observed in T2‐hyperintense WM lesion distribution between patients with impaired and preserved verbal fluency. However, LNM revealed that T2‐hyperintense WM lesions in patients with impaired verbal fluency were more functionally connected to the cerebellum (bilateral cerebellar lobule IX, left cerebellar lobule IV‐V, and right lobule VI) and bilateral thalami (*p* = 0.001, cluster‐wise FWE corrected), as well as to bilateral putamen, caudate nuclei, and right anterior cingulate cortex (*p* = 0.05, cluster‐wise FWE corrected) (Figure 4; Table S9). No significant results were detected for the opposite LNM comparison.

#### 3.2.5 Visual Memory

No significant differences were observed in T2‐hyperintense WM lesion distribution between patients with impaired and preserved visual memory (Figure 4); also, no significant difference in LNM was found between these two patients' subgroups.

### 3.3 T2‐Hyperintense WM LPM and LNM According to Fatigue and Depression

No significant differences in T2‐hyperintense WM lesion distribution and LNM were found in patients with fatigue or depression compared to those without these symptoms (Table S1; Figure S1).

### 3.4 Validation Analysis

LPMs in cognitively preserved, cognitively impaired, non‐fatigued, fatigued, non‐depressed, and depressed MS patients from Scanner 1 and Scanner 2, separately, are reported in Figure S2. There were no systematic differences between lesion maps, suggesting that lesions affected the same WM regions in a comparable way, without any particular regional bias. The Dice coefficient between Scanner 1 and Scanner 2 LPMs was 0.71 in cognitively preserved, 0.77 in cognitively impaired, 0.70 in non‐fatigued, 0.76 in fatigued, 0.73 in non‐depressed, and 0.75 in depressed MS patients, while the MSE between Scanner 1 and Scanner 2 LPMs was 0.0013 in cognitively preserved, 0.0034 in cognitively impaired, 0.002 in non‐fatigued, 0.0035 in fatigued, 0.0027 in non‐depressed, and 0.0029 in depressed MS patients. Overall, these results suggest a relatively high degree of overlap between data from the two scanners.

## 4 Discussion

By applying LNM, we showed that, in MS patients, higher accumulation of T2‐hyperintense WM lesions in cognitively relevant brain areas, along with disrupted intrinsic RS FC with strategic regions, including fronto‐temporo‐parietal cortices, hippocampus, thalamus, and cerebellum, may contribute to both global cognitive impairment and domain‐specific cognitive deficits. Conversely, neither T2‐hyperintense WM lesion distribution nor LNM was associated with fatigue or depression.

In our cohort, mainly comprising relapsing–remitting MS patients with relatively mild disability, 27.2% of patients were classified as cognitively impaired, aligning with prior prevalence estimates of cognitive impairment between 20% and 30%, although higher rates (up to 65%) have also been reported [2, 32]. Information processing speed/attention and verbal memory were the most affected domains, whereas verbal fluency and visual memory were less impaired [2, 32].

Global cognitive impairment was associated with a significantly higher prevalence of T2‐hyperintense WM lesions in several WM tracts, supporting the role of WM lesion‐induced cortical–subcortical disconnection in MS‐related cognitive deficits [2, 3, 4].

LNM further confirmed this, revealing that T2‐hyperintense WM lesions in cognitively impaired patients are more functionally connected to the hippocampi, thalami, cerebellum, and areas of the left occipital cortex.

The thalamus, a central hub interfacing with various brain regions, is essential for information processing speed, attention, verbal fluency, and executive functions. Its structural [33, 34] and RS FC [34, 35, 36] abnormalities have been consistently associated with cognitive impairment in MS. Similarly, hippocampal RS FC abnormalities are associated with cognitive impairment in MS, especially memory deficits [37, 38, 39, 40]. The cerebellum, beyond its role in motor acts, is increasingly recognized for its contribution to cognitive processing [37, 41].

In domain‐specific analysis, lesion distribution and LNMs in patients with impaired information processing speed/attention closely mirrored those observed in patients with global cognitive impairment. Specifically, T2‐hyperintense WM lesions were more widely distributed in several WM tracts and more functionally connected to several GM areas, primarily within deep GM and temporo‐occipital cortices. The similarity of this pattern with that found for global cognitive impairment may reflect the early onset and high prevalence of processing speed and attention deficits, which may represent a key driver of global cognitive dysfunction in our cohort. Moreover, impairment in information processing speed is not only the most frequently affected cognitive domain in MS but also serves as a strong predictor of more global cognitive decline [2, 32]. Specifically, reduced processing speed has been associated with deficits in other cognitive domains, including memory and executive functioning, and is often considered a proxy for global cognitive dysfunction in MS populations [2, 32, 42, 43]. In particular, deficits in information processing speed, as measured by the Symbol Digit Modalities Test (SDMT), are recognized as the most sensitive and clinically relevant indicators of cognitive decline in MS [2, 32, 42, 43]. Clinical guidelines, such as the Brief International Cognitive Assessment for MS (BICAMS) [42], recommend the SDMT as the core measure for routine cognitive screening in MS, due to its strong correlation with overall cognitive functioning.

In verbal memory‐impaired MS patients, T2‐hyperintense WM lesions were more prevalent in the cingulum compared to preserved MS patients and were more functionally connected with the parahippocampal gyri, cerebellum, and temporal poles. These findings highlight the critical role of hippocampal and medial temporal lobe RS FC abnormalities in determining verbal memory impairment [37, 38, 39, 40]. Our results also align with a previous LNM study showing that lesion damage to the hippocampus‐centered brain circuit mediated the association between T2‐hyperintense WM lesion volume and memory deficits in MS patients [19]. Additionally, RS FC abnormalities of the cerebellum [37] may also contribute to explaining verbal memory impairment.

Although verbal fluency impairment was not associated with a significant difference in T2‐hyperintense WM lesion distribution, lesions in verbal fluency‐impaired patients were more functionally connected to thalami, cerebellum, and basal ganglia. This underscores the importance of evaluating lesion connectivity, as T2‐hyperintense WM lesions with similar topographical locations may differ in their functional network impact in key regions, such as the thalamus [34, 35, 36] and cerebellum [37].

Patients with impaired visual memory did not show significant differences in T2‐hyperintense WM lesion distribution or in RS FC profiles, as compared to those with preserved visual memory. These negative findings may reflect a multifactorial nature of visual memory impairment in MS, potentially involving heterogeneous CNS damage not captured by LNM alone, methodological limitations in sensitivity, or a more distributed involvement of neural systems responsible for visual information processing and storage that falls outside the resolution of LNM.

Among the 493 patients assessed for fatigue, 184 (37%) were classified as fatigued, consistent with prior data [8]. Despite a significantly higher T2‐hyperintense WM LV, no specific lesion distribution or RS FC differences with specific brain regions were found. Fatigue in MS has been attributed to dysfunction of cortico‐subcortical networks, especially involving fronto‐parietal cortices and deep GM [6, 7], as well as structural damage, comprising anterior cortico‐subcortical GM atrophy, and lesions and microstructural abnormalities within connecting WM tracts [5, 6, 7, 8]. The lack of significant results in LPM and LNM for fatigue in our cohort may reflect the heterogeneous characteristics of our MS population that included patients with a quite long disease duration and a high prevalence of progressive phenotypes. Indeed, T2‐hyperintense WM lesions in specific locations and their RS FC profile impacting brain regions involved in fatigue may play a more critical role in early disease stages, whereas broader T2‐hyperintense WM lesion accumulation, diffuse normal‐appearing WM damage, and GM volume loss become more prominent over time [6, 7].

Of the 495 patients assessed for depression, 192 (39%) were classified as depressed [1]. However, no differences in T2‐hyperintense WM lesion topography were observed between depressed and non‐depressed patients. Prior studies linking depression to lesions in limbic [9] and association networks (including frontal, temporal, and parietal lobes) [44] have yielded inconsistent results, potentially due to cohort heterogeneity, variable assessment tools, and methodological differences. Lesion‐based approaches have struggled to identify depression‐related topography also in other neurological conditions [45]. A recent study using LNM suggested that depression in MS may involve a specific brain network comprising the medial temporal lobe, the prefrontal cortex (including dorsolateral regions), the retrosplenial cortex, and the intraparietal sulcus [20]. Interestingly, this same circuit is implicated in depression following stroke, traumatic brain injury, and major depression [45, 46]. However, our study did not replicate these findings. Differences in patient phenotype, disability levels, depression assessment tools (MADRS vs. Neuro‐QoL), MRI analyses (whole‐brain vs. circuit‐specific), and statistical design (group comparison vs. correlation with depression scores) may account for this discrepancy [20].

Our study has several limitations. Two different MRI sequences and segmentation techniques for lesion assessment were used; however, scanner distribution was balanced across subgroups, and validation analyses did not indicate major biases. T2‐hyperintense WM lesions represent only one pathological substrate; other factors, including diffuse WM damage and GM atrophy, likely contribute to symptoms with varying effects depending on disease stage. Moreover, even though domain‐specific analyses were conducted independently, due to the concomitant involvement of multiple cognitive domains and the presence of fatigue and depressive symptoms in several MS patients, some results may not be fully specific to a single cognitive domain. Future studies involving more homogeneous patient cohorts are warranted to further investigate the contribution of lesions across different stages of MS and in relation to specific clinical manifestations. This was a cross‐sectional study. Longitudinal data would better elucidate how T2‐hyperintense WM lesion accumulation and connectivity changes may contribute to evolving clinical manifestations. We used the MFIS and MADRS scales, both validated in MS populations, but these tools may not fully capture the temporal dynamics of fatigue. Moreover, their scores may partially correlate with patients' cognitive outcomes, possibly leading to a partial loss of specificity of some results. Functional MRI fluctuations have a relatively low signal‐to‐noise ratio in WM, making the application of the LNM approach not optimally suited for MS. However, to mitigate this, similar to previous works [19, 20], we used a large human connectome (*n* = 1000), which enhanced RS FC reliability for seeds located in WM. Moreover, we recruited a large cohort of patients, since a high sample size is thought to provide enough signal to overcome the noise [20], even at the cost of combining lesion masks derived from different MRI sequences. In addition, it has been recently shown that fMRI signal of WM is likely to represent tract‐specific responses to neural activity, supporting the rationale of utilizing WM lesions in connectivity analysis [47]. Finally, we relied on a normative database of healthy controls to estimate RS FC for T2‐hyperintense WM lesions, as previously done [19, 20]. While it has been already demonstrated that LNM methodology is robust across different normative datasets with different age distributions [14], connectivity measurements specific for each patient could potentially improve the associations between lesions and clinical manifestations, but this may introduce additional noise into the analysis.

In conclusion, regional accumulation of T2‐hyperintense WM lesions may disrupt intrinsic RS FC with key brain regions such as the hippocampus, thalamus, cerebellum, and temporo‐occipital cortices, thereby contributing to cognitive impairment in MS. Future studies integrating structural connectivity LNM (e.g., using diffusion‐weighted MRI) with functional LNM could provide multimodal corroboration of the mechanisms underlying heterogeneous cognitive impairments in this disease.

## Author Contributions

P.P., M.F., and M.A.R. contributed to the conception and design of the study. A.F., P.P., P.V., D.M., M.M., F.E., M.F., and M.A.R. contributed to the acquisition and analysis of data. A.F., P.P., P.V., D.M., M.M., F.E., M.F., and M.A.R. contributed to drafting the text and preparing the figures. A.F., P.P., P.V., D.M., M.M., F.E., M.F., and M.A.R. approved the final draft of the manuscript.

## Conflicts of Interest

The authors declare no conflicts of interest. Potential conflicts of interest outside the submitted work are as follows: A. Franceschini has nothing to disclose. P. Preziosa received speaker honoraria from Roche, Biogen, Novartis, Merck, Bristol Myers Squibb, Genzyme, Horizon, and Sanofi; he has received research support from the Italian Ministry of Health and Fondazione Italiana Sclerosi Multipla. P. Valsasina has nothing to disclose. D. Mistri has nothing to disclose. M. Margoni reports grants and personal fees from Sanofi Genzyme, Merck Serono, Roche, Biogen, and Novartis. F. Esposito has nothing to disclose. M. Filippi is Editor‐in‐Chief of the Journal of Neurology, Associate Editor of Human Brain Mapping, Neurological Sciences, and Radiology; received compensation for consulting services from Alexion, Almirall, Biogen, Merck, Novartis, Roche, and Sanofi; speaking activities from Bayer, Biogen, Celgene, Chiesi Italia SpA, Eli Lilly, Genzyme, Janssen, Merck‐Serono, Neopharmed Gentili, Novartis, Novo Nordisk, Roche, Sanofi, Takeda, and TEVA; participation in Advisory Boards for Alexion, Biogen, Bristol‐Myers Squibb, Merck, Novartis, Roche, Sanofi, Sanofi‐Aventis, Sanofi‐Genzyme, and Takeda; scientific direction of educational events for Biogen, Merck, Roche, Celgene, Bristol‐Myers Squibb, Lilly, Novartis, and Sanofi‐Genzyme; he receives research support from Biogen Idec, Merck‐Serono, Novartis, Roche, the Italian Ministry of Health, the Italian Ministry of University and Research, and Fondazione Italiana Sclerosi Multipla. M.A. Rocca received consulting fees from Biogen, Bristol Myers Squibb, and Roche; and speaker honoraria from Alexion, Biogen, Bristol Myers Squibb, Celgene, Horizon Therapeutics Italy, Merck Serono SpA, Mitsubishi‐Tanabe Pharma, Neuraxpharm, Novartis, Roche, Sandoz, and Sanofi. She receives research support from the MS Society of Canada, the Italian Ministry of Health, the Italian Ministry of University and Research, and Fondazione Italiana Sclerosi Multipla. She is Associate Editor for *Multiple Sclerosis and Related Disorders* and Associate Co‐Editor for Europe and Africa for *Multiple Sclerosis Journal*.

## Supporting information


**Figure S1:** (A) LPMs of MS patients with (in red, *N* = 184) and without (in cyan, *N* = 309) fatigue. (B) LPMs of MS patients with (in red, *N* = 192) and without (in cyan, *N* = 303) depression.
**Figure S2:** (A) LPMs of cognitively preserved (CP) and cognitively impaired (CI) MS patients from Scanner 1 and Scanner 2. In red, LPMs of CP/CI patients acquired on Scanner 1 (*N* = 237/68, respectively); in blue, LPMs of CP/CI patients acquired on Scanner 2 (*N* = 197/94). In purple, regions where LPMs of Scanner 1 and Scanner 2 overlay. (B) LPMs of non‐fatigued (N‐FAT) and fatigued (FAT) MS patients from Scanner 1 and Scanner 2. In red, LPMs of N‐FAT/FAT patients acquired on Scanner 1 (*N* = 178/94, respectively); in blue, LPMs of N‐FAT/FAT patients acquired on Scanner 2 (*N* = 131/90). In purple, regions where LPMs of Scanner 1 and Scanner 2 overlay. (C) LPMs of non‐depressed (ND) and depressed (D) MS patients from Scanner 1 and Scanner 2. In red, LPMs of ND/D patients acquired on Scanner 1 (*N* = 188/79, respectively); in blue, LPMs of ND/D patients acquired on Scanner 2 (*N* = 115/113). In purple, regions where LPMs of Scanner 1 and Scanner 2 overlay.
**Table S1:** Main demographic, clinical and neuropsychological features of multiple sclerosis (MS) patients.
**Table S2:** Demographic, clinical, and MRI features of patients with and without fatigue or depression.
**Table S3:** Results of the comparison of lesion distribution, showing a higher prevalence of T2‐hyperintense WM lesions in cognitively impaired compared to cognitively preserved MS patients for global cognitive function (between‐group comparison performed using statistical non‐parametric mapping [SnPM] toolbox, scanner‐, age‐, EDSS‐ and sex‐adjusted, *p* < 0.05 family‐wise error corrected for multiple comparisons, cluster extent *k*_E_ ≥ 50). JHU_WM_tractography atlas was used for description of WM lesion location. Fiber tracts involved at more or equal than 5% are reported.
**Table S4:**. Results of the comparison of lesion network maps (LNMs), showing clusters presenting with higher resting state functional connectivity (RS FC) with T2‐hyperintense WM lesions in cognitively impaired compared to cognitively preserved MS patients (SPM12 scanner‐, age‐, EDSS‐, T2 lesion volume and sex‐adjusted full factorial model, *p* < 0.05 FWE corrected for multiple comparisons ‐cluster forming threshold: *p* < 0.001, uncorrected, cluster extent *k*_E_ ≥ 50). AAL atlas was used for description of GM regions location.
**Table S5:** Results of the comparison of lesion distribution, showing a higher prevalence of T2‐hyperintense WM lesion distribution in MS patients with impaired compared to preserved information processing speed/attention (between‐group comparison performed using statistical non‐parametric mapping [SnPM] toolbox, scanner‐, age‐, EDSS‐ and sex‐adjusted, *p* < 0.05 family‐wise error corrected for multiple comparisons, cluster extent *k*_E_ ≥ 50). JHU_WM_tractography atlas was used for description of WM lesion location. Fiber tracts involved at more or equal than 5% are reported.
**Table S6:** Results of the comparison of lesion network maps (LNMs), showing clusters presenting with higher resting state functional connectivity (RS FC) with T2‐hyperintense WM lesions in MS patients with impaired compared to preserved information processing speed/attention (SPM12 scanner‐, age‐, EDSS‐, T2 lesion volume and sex‐adjusted full factorial model, *p* < 0.05 FWE corrected for multiple comparisons ‐cluster forming threshold: *p* < 0.001, uncorrected, cluster extent *k*_E_ ≥ 50). AAL atlas was used for description of GM regions location.
**Table S7:** Results of the comparison of lesion distribution, showing a higher prevalence of T2‐hyperintense WM lesions in MS patients with impaired compared to preserved verbal memory (between‐group comparison performed using statistical non‐parametric mapping [SnPM] toolbox, scanner‐, age‐, EDSS‐ and sex‐adjusted, *p* < 0.05 family‐wise error corrected for multiple comparisons, cluster extent *k*_E_ ≥ 50). JHU_WM_tractography atlas was used for description of WM lesion location. Fiber tracts involved at more or equal than 5% are reported.
**Table S8:** Results of the comparison of lesion network maps (LNMs), showing clusters presenting with higher resting state functional connectivity (RS FC) with T2‐hyperintense WM lesions in patients with impaired compared to preserved verbal memory (SPM12 scanner‐, age‐, EDSS‐, T2 lesion volume and sex‐adjusted full factorial model, *p* < 0.05 FWE corrected for multiple comparisons ‐cluster forming threshold: *p* < 0.001, uncorrected, cluster extent *k*_E_ ≥ 50). AAL atlas was used for description of GM regions location.
**Table S9:** Results of the comparison of lesion network maps (LNMs), showing clusters presenting with higher resting state functional connectivity (RS FC) with T2‐hyperintense WM lesions in patients with impaired compared to preserved verbal fluency (SPM12 scanner‐, age‐, EDSS‐, T2 lesion volume and sex‐adjusted full factorial model, *p* < 0.05 FWE corrected for multiple comparisons ‐cluster forming threshold: *p* < 0.001, uncorrected, cluster extent *k*_E_ ≥ 50). AAL atlas was used for description of GM regions location.

## Data Availability

The corresponding author, who had complete access to all the data of the study, assumes responsibility for the integrity of the data and accuracy in the analysis. The anonymized data set used and analyzed for this study can be obtained from the corresponding author on reasonable request.
