# Supplementary material for: Lesion Location and Functional Connections Reveal Cognitive Impairment Networks in Multiple Sclerosis
Source: Ann Clin Transl Neurol. 2025 Oct 5;13(2):242–55. doi: 10.1002/acn3.70199 (PMC12883681; doi:10.1002/acn3.70199)
Supplement: Supplementary file 1 — Figure S1: (A) LPMs of MS patients with (in red, N = 184) and without (in cyan, N = 309) fatigue. (B) LPMs of MS patients with (in red, N = 192) and without (in cyan, N = 303) depression. Figure S2: (A) LPMs of cognitively preserved (CP) and cognitively impaired (CI) MS patients from Scanner 1 and Scanner 2. In red, LPMs of CP/CI patients acquired on Scanner 1 (N = 237/68, respectively); in blue, LPMs of CP/CI patients acquired on Scanner 2 (N = 197/94). In purple, regions where LPMs of Scanner 1 and Scanner 2 overlay. (B) LPMs of non‐fatigued (N‐FAT) and fatigued (FAT) MS patients from Scanner 1 and Scanner 2. In red, LPMs of N‐FAT/FAT patients acquired on Scanner 1 (N = 178/94, respectively); in blue, LPMs of N‐FAT/FAT patients acquired on Scanner 2 (N = 131/90). In purple, regions where LPMs of Scanner 1 and Scanner 2 overlay. (C) LPMs of non‐depressed (ND) and depressed (D) MS patients from Scanner 1 and Scanner 2. In red, LPMs of ND/D patients acquired on Scanner 1 (N = 188/79, respectively); in blue, LPMs of ND/D patients acquired on Scanner 2 (N = 115/113). In purple, regions where LPMs of Scanner 1 and Scanner 2 overlay. Table S1: Main demographic, clinical and neuropsychological features of multiple sclerosis (MS) patients. Table S2: Demographic, clinical, and MRI features of patients with and without fatigue or depression. Table S3: Results of the comparison of lesion distribution, showing a higher prevalence of T2‐hyperintense WM lesions in cognitively impaired compared to cognitively preserved MS patients for global cognitive function (between‐group comparison performed using statistical non‐parametric mapping [SnPM] toolbox, scanner‐, age‐, EDSS‐ and sex‐adjusted, p < 0.05 family‐wise error corrected for multiple comparisons, cluster extent k E ≥ 50). JHU_WM_tractography atlas was used for description of WM lesion location. Fiber tracts involved at more or equal than 5% are reported. Table S4:. Results of the comparison of lesion network maps (LNMs), sh [file ACN3-13-242-s001.docx]

**Supplementary material**

**Supplementary Table 1.** Main demographic, clinical and neuropsychological features of multiple sclerosis (MS) patients.

| **Variables** | | **MS patients (n=596)** |
| --- | --- | --- |
| **Sex (%): Women/Men** | | 350 (59) / 246 (41) |
| **Mean age (SD) [years]** | | 43.7 (11.5) |
| **Scanner 1/2** | | 305/291 |
| **Mean disease duration (SD) [years]** | | 13.1 (9.8) |
| **Median EDSS score (IQR)** | | 2.5 (1.5;5.5) |
| **Clinical phenotype (%): RRMS/PMS** | | 383 (64) / 213 (36) |
| **Ongoing DMT^a^ (%): none/first line/second line** | | 134 (22) /242 (41) / 220 (37) |
| **Median education (IQR) [years]** | | 13.0 (9.0;17.8) |
| **Number (%) of preserved / impaired MS patients** | **Global cognition** | 434 (73) /162 (27) |
|  | **Verbal memory** | 416 (70) /180 (30) |
|  | **Visuo-spatial learning** | 494 (83) /102 (17) |
|  | **Information processing speed/attention** | 384 (64) /212 (36) |
|  | **Verbal fluency^b^** | 471 (83) /97 (17) |
| **Mean MFIS^c^ (SD)** | | 32.1 (17.6) |
| **Number (%) of subjects with fatigue^c^** | | 184 (37) |
| **Mean MADRS^d^ (SD)** | | 9.2 (7.6) |
| **Number (%) of subjects with depression^d^** | | 192 (39) |
| **Median T2-hyperintense WM LV (IQR) [ml]** | | 4.3 (1.5;10.1) |
| **Mean NBV (SD) [ml]** | | 1526 (79) |

Abbreviations: DMT=disease-modifying therapy; EDSS=Expanded Disability Status Scale; IQR=interquartile range; LV=lesion volume; ml=milliliters; MADRS=Montgomery-Åsberg Depression Rating Scale; MFIS=Modified form of the fatigue Impact Scale; NBV=normal brain volume; P=preserved; PMS=progressive multiple sclerosis; RRMS=relapsing–remitting multiple; SE=standard error; SD=standard deviation; WM=white matter.

^a^ First line=glatiramer acetate, interferon beta 1a, teriflunomide or dimethyl fumarate; Second line=fingolimod, siponimod, ozanimod, natalizumab, cladribine, anti-CD20 (ocrelizumab, rituximab, ofatumumab) and other immunosuppressants.

^b^Available for 568 out of 596 MS patients.

^c^Available for 493 out of 596 MS patients.

^d^Available for 495 out of 596 MS patients.

**Supplementary Table 2.** Demographic, clinical, and MRI features of patients with and without fatigue or depression.

| **Variables** | **Fatigue^a^ (n=493)** | | | **Depression^b^ (n=495)** | | |
| --- | --- | --- | --- | --- | --- | --- |
|  | **MS patients without fatigue**  **(n=309)** | **MS patients with fatigue**  **(n=184)** | **p** | **MS patients without depression**  **(n=303)** | **MS patients with depression**  **(n=192)** | **p** |
| **Sex (%): Women/Men** | 166 (54)/143 (46) | 115 (63)/69 (37) | 0.057^c^ | 161 (53)/ 142 (47) | 122 (64)/ 70 (36) | **0.023^c^** |
| **Mean age (SD) [years]** | 42.3 (11.1) | 46.3 (11.4) | **<0.001^d^** | 43.1 (10.8) | 44.9 (11.8) | 0.087^d^ |
| **Scanner 1/2** | 178/131 | 94/90 | 0.16^c^ | 188/115 | 79/113 | **0.001^c^** |
| **Mean disease duration (SD) [years]** | 12.1 (9.4) | 14.6 (9.9) | **0.005^d^** | 12.6 (9.6) | 13.9 (10.0) | 0.151^d^ |
| **Median EDSS score (IQR)** | 1.5 (1.3;4.0) | 5.0 (2.5;6.5) | **<0.001^e^** | 2.0 (1.5;4.5) | 3.5 (1.5;6.0) | **<0.001^e^** |
| **Clinical phenotype (%): RRMS/PMS** | 236 (76)/73 (24) | 82 (45)/102 (55) | **<0.001^c^** | 210 (69)/ 93 (31) | 115 (60)/ 77 (40) | **0.032^c^** |
| **Ongoing DMT (%): none/first line/second line^f^** | 51 (17)/131 (42)/127 (41) | 43 (23)/65 (35)/76 (41) | 0.116**^c^** | 61 (20)/119 (39)/ 123 (41) | 41 (21)/76 (40)/ 75 (39) | 0.925**^c^** |
| **Median education (IQR) [years]** | 13.0 (11.0;18.0) | 13.0 (9.3;18.0) | 0.908^e^ | 13.0 (11.4;17.0) | 13.0 (8.0;18.0) | 0.152**^e^** |
| **Mean MFIS (SD)** | 21.0 (10.4) | 50.8 (9.7) | **<0.001^d^** | 25.3 (15.2) | 42.0 (15.4) | **<0.001^d^** |
| **Mean MADRS (SD)** | 6.5 (5.5) | 14.1 (8.5) | **<0.001^d^** | 4.4 (2.8) | 16.9 (6.3) | **<0.001^d^** |
| **Median T2-hyperintense LV (IQR) [ml]^g^** | 3.3 (1.2;7.3) | 5.6 (2.1;14.7) | **<0.001^h^** | 3.4 (1.2;8.7) | 5.2 (2.0;11.0) | 0.076 ^h^ |
| **Estimated mean NBV (SE) [ml]** | 1533 (4) | 1515 (5) | **0.005^h^** | 1525 (4) | 1528 (5) | 0.673^h^ |

Abbreviations: DMT=disease modifying therapy; EDSS=Expanded Disability Status Scale; IQR=interquartile range; LV=lesion volume; MADRS=Montgomery-Åsberg Depression Rating Scale; MFIS=Modified form of the Fatigue Impact Scale; ml=milliliters; NBV=normal brain volume; PMS=progressive multiple sclerosis; RRMS=relapsing–remitting multiple; SD=standard deviation; SE=standard error.

^a^Available for 493 out of 596 MS patients.

^b^Available for 495 out of 596 MS patients.

^c^Chi-square test.

^d^Two sample *t* test.

^e^Mann-Whitney U test.

^f^ First line=glatiramer acetate, interferon beta 1a, teriflunomide or dimethyl fumarate; Second line = fingolimod, siponimod, ozanimod, natalizumab, cladribine, anti-CD20 (ocrelizumab, rituximab, ofatumumab) and other immunosuppressants.

^g^Comparison performed on log-scale.

^h^Age-adjusted, scanner-adjusted and sex-adjusted linear regression model.

Bold text indicates a statistically significant result.

**Supplementary Table 3.** Results of the comparison of lesion distribution, showing a higher prevalence of T2-hyperintense WM lesions in cognitively impaired compared to cognitively preserved MS patients for global cognitive function (between-group comparison performed using statistical non-parametric mapping [SnPM] toolbox, scanner-, age-, EDSS- and sex-adjusted, p<0.05 family-wise error corrected for multiple comparisons, cluster extent k_E_ >50). JHU_WM_tractography atlas was used for description of WM lesion location. Fiber tracts involved at more or equal than 5% are reported.

| **WM lesion location** | **MNI space coordinates (x, y, z)** | **T value** | **K_E_** | **p (FWE-corrected)** |
| --- | --- | --- | --- | --- |
| L Corpus callosum, body | -16 0 30  -12 6 24 | 8.03  5.32 | 76 | 0.0002 |
| L Superior longitudinal fasciculus  L Posterior corona radiata | 28 -32 30  28 -36 22 | 7.09  6.17 | 59 | 0.0002 |
| R Internal capsule  R Posterior thalamic radiation  R Inferior longitudinal fasciculus | -32 -36 4  -34 -46 2  -38 -42 -8 | 6.81  6.07  6.04 | 51 | 0.0002 |
| L Anterior corona radiata  L Corpus callosum, body | -22 16 24  -14 12 18 | 6.46  5.71 | 50 | 0.0002 |
| L Posterior thalamic radiation | -28 -68 12  -22 -74 14 | 6.52  5.32 | 50 | 0.0002 |

Abbreviations: EDSS=Expanded Disability Status Scale; FEW=family-wise error; JHU=Johns Hopkins University; L=left; MNI=Montreal Neurosciences Institute; MS=multiple sclerosis; R=right; SPM=Statistical Parametric Mapping; WM=white matter.

**Supplementary Table 4.** Results of the comparison of lesion network maps (LNMs), showing clusters presenting with higher resting state functional connectivity (RS FC) with T2-hyperintense WM lesions in cognitively impaired compared to cognitively preserved MS patients (SPM12 scanner-, age-, EDSS-, T2 lesion volume and sex-adjusted full factorial model, p<0.05 FWE corrected for multiple comparisons -cluster forming threshold: p<0.001, uncorrected, cluster extent k_E_ >50). AAL atlas was used for description of GM regions location.

| **Connected regions** | **MNI space coordinates (x, y, z)** | **T value** | **K_E_** | **p (FWE-corrected)** |
| --- | --- | --- | --- | --- |
| L thalamus | -20 -28 8 | 4.47 | 150 | 0.05 |
| R cerebellum (lobule VI)  L cerebellum (lobule VI) | 12 -68 22  -8 -64 20 | 4.18  4.13 | 251 | 0.05 |
| R thalamus | 18 -24 10 | 4.07 | 53 | 0.05 |
| R hippocampus | 22 -30 14 | 4.02 | 51 | 0.05 |
| L hippocampus | -22 -38 2 | 3.99 | 50 | 0.05 |
| L lingual gyrus | -26 -44 -2 | 3.97 | 50 | 0.05 |

Abbreviations: AAL=Automatic Anatomical Labelling; EDSS=Expanded Disability Status Scale; FWE=family-wise error; GM=gray matter; L=left; MNI=Montreal Neurosciences Institute; R=right; SPM=Statistical Parametric Mapping; WM=white matter.

**Supplementary Table 5.** Results of the comparison of lesion distribution, showing a higher prevalence of T2-hyperintense WM lesion distribution in MS patients with impaired compared to preserved information processing speed/attention (between-group comparison performed using statistical non-parametric mapping [SnPM] toolbox, scanner-, age-, EDSS- and sex-adjusted, p<0.05 family-wise error corrected for multiple comparisons, cluster extent k_E_ >50). JHU_WM_tractography atlas was used for description of WM lesion location. Fiber tracts involved at more or equal than 5% are reported.

| **Connected regions** | **MNI space coordinates (x, y, z)** | **T value** | **K_E_** | **p (FWE-corrected)** |
| --- | --- | --- | --- | --- |
| L Superior corona radiata  L Superior fronto-occipital fasciculus | -20 4 30  -16 0 24 | 7.46  5.88 | 85 | 0.0002 |
| L Corpus callosum, splenium  L Posterior thalamic radiation  L Posterior thalamic radiation | -18 -44 28  -28 -68 12  -34 -56 16 | 7.21  6.73  6.50 | 375 | 0.0002 |
| L Posterior thalamic radiation  L Inferior longitudinal fasciculus  L Inferior longitudinal fasciculus | -34 -52 6  -36 -36 -4  -34 -46 -4 | 6.93  6.07  5.99 | 68 | 0.0002 |
| R Superior corona radiata  R corpus callosum, body  R Superior fronto-occipital fasciculus | 18 -4 26  20 6 30  16 4 22 | 6.67  5.92  5.71 | 84 | 0.0002 |
| L Corpus callosum, genu  L Anterior corona radiata | -20 26 8  -20 28 16 | 6.29  5.99 | 50 | 0.0004 |
| R Posterior thalamic radiation  R Corpus callosum, splenium  R Posterior thalamic radiation | 28 -64 14  26 -72 10  30 -64 6 | 6.28  5.89  5.49 | 53 | 0.0004 |

Abbreviations: EDSS=Expanded Disability Status Scale; FWE=family-wise error; JHU=Johns Hopkins University; L=left; MNI=Montreal Neurosciences Institute; MS=multiple sclerosis; R=right; SPM=Statistical Parametric Mapping; WM=white matter.

**Supplementary Table 6.** Results of the comparison of lesion network maps (LNMs), showing clusters presenting with higher resting state functional connectivity (RS FC) with T2-hyperintense WM lesions in MS patients with impaired compared to preserved information processing speed/attention (SPM12 scanner-, age-, EDSS-, T2 lesion volume and sex-adjusted full factorial model, p<0.05 FWE corrected for multiple comparisons -cluster forming threshold: p<0.001, uncorrected, cluster extent k_E_ >50). AAL atlas was used for description of GM regions location.

| **Connected regions** | **MNI space coordinates (x, y, z)** | **T value** | **K_E_** | **p (FWE-corrected)** |
| --- | --- | --- | --- | --- |
| R hippocampus  R thalamus | 24 -34 6  18 -28 6 | 4.66  4.27 | 78 | 0.05 |
| L thalamus  R hippocampus | -20 -30 6  -22 -38 2 | 4.47  3.93 | 103 | 0.05 |
| L lingual gyrus  R lingual gyrus  L fusiform gyrus | -28 -46 -4  0 -74 2  -30 -68 -4 | 4.09  4.06  3.97 | 3704 | 0.01 |
| R parahippocampal gyrus | 28 -42 -4 | 4.07 | 53 | 0.05 |
| R fusiform gyrus | 30 -58 -12 | 3.99 | 50 | 0.05 |

Abbreviations: AAL=Automatic Anatomical Labelling; EDSS=Expanded Disability Status Scale; FWE=family-wise error; GM=gray matter; L=left; MNI=Montreal Neurosciences Institute; R=right; SPM= Statistical Parametric Mapping; WM=white matter.

**Supplementary Table 7.** Results of the comparison of lesion distribution, showing a higher prevalence of T2-hyperintense WM lesions in MS patients with impaired compared to preserved verbal memory (between-group comparison performed using statistical non-parametric mapping [SnPM] toolbox, scanner-, age-, EDSS- and sex-adjusted, p<0.05 family-wise error corrected for multiple comparisons, cluster extent k_E_ >50). JHU_WM_tractography atlas was used for description of WM lesion location. Fiber tracts involved at more or equal than 5% are reported.

| **WM lesion location** | **MNI space coordinates (x, y, z)** | **T value** | **K_E_** | **p (FWE-corrected)** |
| --- | --- | --- | --- | --- |
| L Cingulum | -36 -36 -4 | 6.13 | 50 | 0.0008 |

Abbreviations: EDSS=Expanded Disability Status Scale; FWE=family-wise error; JHU=Johns Hopkins University; L=left; MNI=Montreal Neurosciences Institute; MS=multiple sclerosis; R=right; SPM= Statistical Parametric Mapping; WM=white matter.

**Supplementary Table 8**. Results of the comparison of lesion network maps (LNMs), showing clusters presenting with higher resting state functional connectivity (RS FC) with T2-hyperintense WM lesions in patients with impaired compared to preserved verbal memory (SPM12 scanner-, age-, EDSS-, T2 lesion volume and sex-adjusted full factorial model, p<0.05 FWE corrected for multiple comparisons -cluster forming threshold: p<0.001, uncorrected, cluster extent k_E_ >50). AAL atlas was used for description of GM regions location.

| **Connected regions** | **MNI space coordinates (x, y, z)** | **T value** | **K_E_** | **p (FWE-corrected)** |
| --- | --- | --- | --- | --- |
| R cerebellum (lobule VIII) | 32 -58 -54 | 4.42 | 83 | 0.05 |
| L temporal pole | -18 4 -36 | 4.17 | 118 | 0.05 |
| R cerebellum (lobule IV-V) | 26 -34 -26 | 4.07 | 73 | 0.05 |
| L parahippocampal gyrus | -36 -44 -12 | 3.99 | 78 | 0.05 |
| L cerebellum (lobule IV-V) | -28 -34 -28 | 4.02 | 53 | 0.05 |
| R cerebellum (lobule VI) | 16 -66 -24 | 3.97 | 56 | 0.05 |
| R temporal pole | 46 4 -16 | 3.96 | 50 | 0.05 |

Abbreviations: AAL=Automatic Anatomical Labelling; EDSS=Expanded Disability Status Scale; FWE=family-wise error; GM=gray matter; L=left; MNI=Montreal Neurosciences Institute; R=right; SPM= Statistical Parametric Mapping; WM=white matter.

**Supplementary Table 9.** Results of the comparison of lesion network maps (LNMs), showing clusters presenting with higher resting state functional connectivity (RS FC) with T2-hyperintense WM lesions in patients with impaired compared to preserved verbal fluency (SPM12 scanner-, age-, EDSS-, T2 lesion volume and sex-adjusted full factorial model, p<0.05 FWE corrected for multiple comparisons -cluster forming threshold: p<0.001, uncorrected, cluster extent k_E_ >50). AAL atlas was used for description of GM regions location.

| **Connected regions** | **MNI space coordinates (x, y, z)** | **T value** | **K_E_** | **p (FWE-corrected)** |
| --- | --- | --- | --- | --- |
| L thalamus  R thalamus  R cerebellum (lobule IX)  L cerebellum (lobule IX)  R cerebellum (lobule VI)  L cerebellum (lobule IV-V) | -20 -28 10  22 -30 14  12 -46 -54  -8 -46 -54  32 -32 -32  -26 -30 -34 | 4.69  4.14  4.23  4.14  3.92  3.89 | 8799 | 0.001 |
| R putamen | 28 6 8 | 4.37 | 204 | 0.05 |
| L putamen | -30 2 2 | 4.09 | 292 | 0.05 |
| R caudate | 10 14 12 | 4.03 | 123 | 0.05 |
| L caudate | -14 20 2 | 4.00 | 50 | 0.05 |
| R anterior cingulate cortex | 0 32 4 | 3.97 | 50 | 0.05 |

Abbreviations: AAL=Automatic Anatomical Labelling; EDSS=Expanded Disability Status Scale; FWE=family-wise error; GM=gray matter; L=Left; MNI=Montreal Neurosciences Institute; R=right; SPM=Statistical Parametric Mapping; WM=white matter.


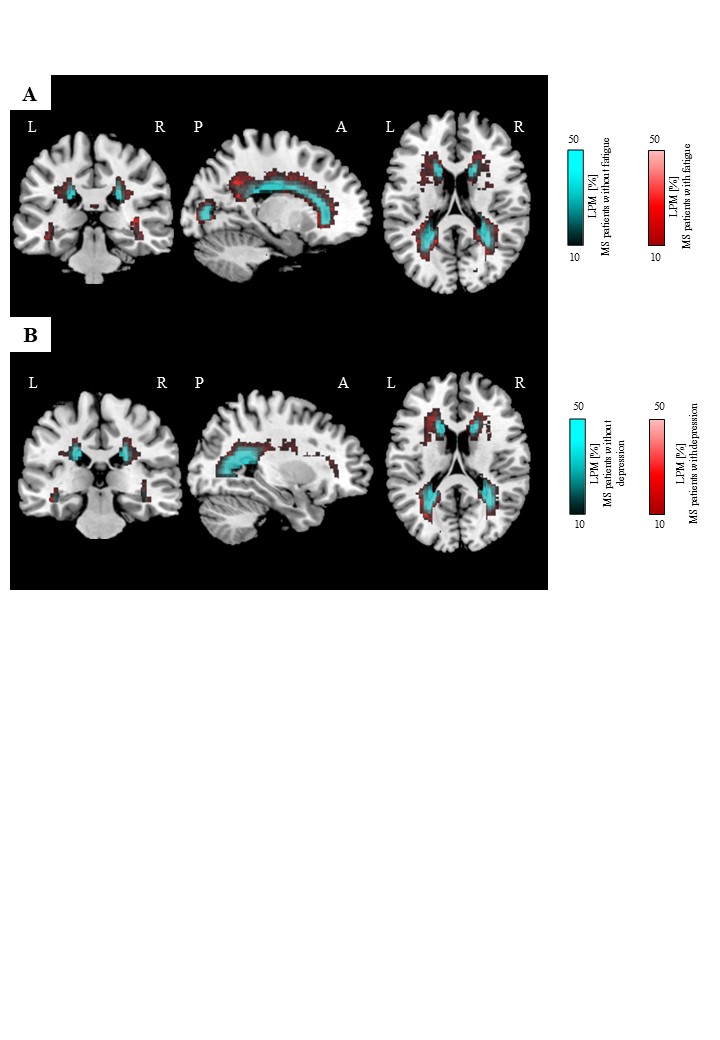


**Supplementary Figure 1.** A) LPMs of MS patients with (in red, N=184) and without (in cyan, N=309) fatigue. B) LPMs of MS patients with (in red, N=192) and without (in cyan, N=303) depression.

Abbreviations: A=anterior; L=left; LPM=lesion probability map; MS=multiple sclerosis; P=posterior and R=right.


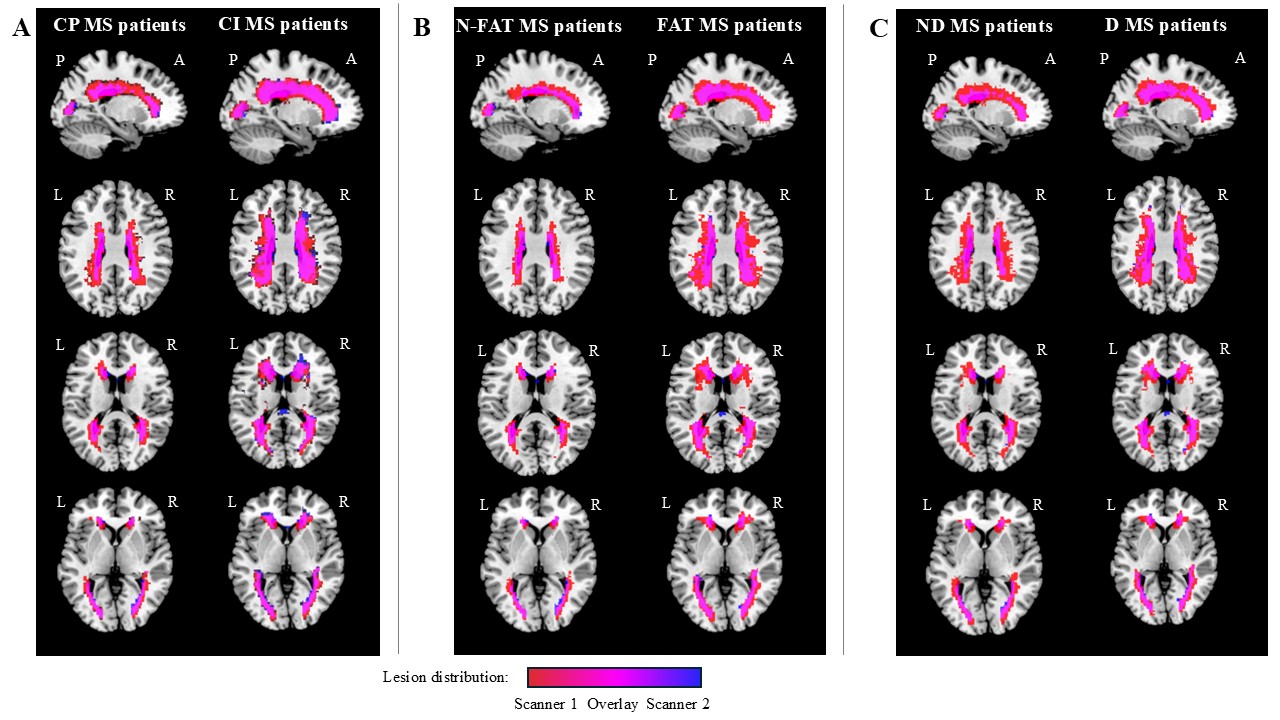


**Supplementary Figure 2.** A) LPMs of cognitively preserved (CP) and cognitively impaired (CI) MS patients from Scanner 1 and Scanner 2. In red, LPMs of CP/CI patients acquired on Scanner 1 (N=237/68, respectively); in blue, LPMs of CP/CI patients acquired on Scanner 2 (N=197/94). In purple, regions where LPMs of Scanner 1 and Scanner 2 overlay. B) LPMs of non-fatigued (N-FAT) and fatigued (FAT) MS patients from Scanner 1 and Scanner 2. In red, LPMs of N-FAT/FAT patients acquired on Scanner 1 (N=178/94, respectively); in blue, LPMs of N-FAT/FAT patients acquired on Scanner 2 (N=131/90). In purple, regions where LPMs of Scanner 1 and Scanner 2 overlay. C) LPMs of non-depressed (ND) and depressed (D) MS patients from Scanner 1 and Scanner 2. In red, LPMs of ND/D patients acquired on Scanner 1 (N=188/79, respectively); in blue, LPMs of ND/D patients acquired on Scanner 2 (N=115/113). In purple, regions where LPMs of Scanner 1 and Scanner 2 overlay.

Abbreviations: A=anterior; L=left; LPM=lesion probability map; MS=multiple sclerosis; P=posterior and R=right.
